# Excess Enthalpies Analysis of Biofuel Components: Sunflower Oil–Alcohols Systems

**DOI:** 10.3390/ijms25063244

**Published:** 2024-03-13

**Authors:** Alexandra Golikova, Anna Shasherina, Yuri Anufrikov, Georgii Misikov, Petr Kuzmenko, Alexander Smirnov, Maria Toikka, Alexander Toikka

**Affiliations:** Institute of Chemistry, St. Petersburg State University, Universitetskiy Prospect 26, Peterhof, Saint Petersburg 198504, Russia; annakirillova980@mail.ru (A.S.); anufrikov_yuri@mail.ru (Y.A.); zakgeor@list.ru (G.M.); piter8101993@mail.ru (P.K.); a.a.smirnov97@yandex.ru (A.S.); m.toikka@spbu.ru (M.T.); toikka@yandex.ru (A.T.)

**Keywords:** excess enthalpy, calorimetry, sunflower oil, thermochemistry, Redlich–Kister equation, NRTL

## Abstract

This study addresses the pressing issues of energy production and consumption, in line with global sustainable development goals. Focusing on the potential of alcohols as “green” alternatives to traditional fossil fuels, especially in biofuel applications, we investigate the thermochemical properties of three alcohols (n-propanol, n-butanol, n-pentanol) blended with sunflower oil. The calorimetric analysis allows for the experimental determination of excess enthalpies in pseudo-binary mixtures at 303.15 K, revealing similarities in the trends of the curves (dependence on concentrations) but with different values for the excess enthalpies for each mixture. Despite the structural differences of the alcohols studied, the molar excess enthalpy values exhibit uniformity, suggesting consistent mixing behavior. The peak values of excess enthalpies for systems with sunflower oil and n-propanol, n-butanol and n-pentanol are, respectively, 3255.2 J/mole, 3297.4 J/mole and 3150.1 J/mole. Both the NRTL and Redlich–Kister equations show satisfactory agreement with the obtained values.

## 1. Introduction

It is a matter of common observation that the problems of energy production and consumption remain some of the most acute in the modern world, for both industry and society. The global attention paid to the use of traditional energy sources and development of new ones is reflected in 2 (the 7th and 12th) of the 17 Sustainable Development Goals that were proposed in the 2030 Agenda for Sustainable Development by the United Nations General Assembly in 2015 [1]. Such a high level of interest in obtaining “affordable” and “green” energy resulted in extensive scientific research and discussions on developing alternative ways to produce energy all over the world, e.g., perspectives on using biodiesel in Turkey [2], India [3], the USA [4] and in Latin America [5] have been discussed. Various biofuels, which can be produced from natural resources, are believed to be effective alternatives to the fossil fuels. Cherwoo et al. [6] discussed different aspects of the biofuel industry; in particular, three generations of biofuels were observed with their advantages and drawbacks that restrict their wide commercial usage.

Alcohols are promising “green” alternatives to the traditional fossil fuels. Though bioethanol is the most widely used one, other alcohols, mainly C1–C5, are also of scientific and commercial interest. At the same time, alcohols can also be applied as additives to biodiesel to enhance its properties. Manivasagam et al. [7] studied the influence of propanol electronic mode fumigation on the performance and emission characteristics of compression ignition engines with diesel and lemongrass oil biodiesel. Propanol fumigation has been found to decrease the level of smoke and carbon dioxide emissions. Tosun and Aydin [8] investigated the properties of binary and ternary blends of propanol with diesel fuel and biodiesel from safflower oil. It has been observed that the addition of alcohol has a positive impact on viscosity, mixing and evaporation properties as well as the thermal efficiency of the blended fuel. The influence of propanol on diesel and biodiesel (from cooking oil and Ambadi seed oil) was observed in paper [9]. The mixtures with the alcohol showed better brake efficiency, lower emissions with higher cylinder pressure and a peak heat release rate when tested in terms of engine characteristics. Musthafa et al. [10] also discovered that the main advantage of adding alcohols (in particular ethanol, propanol and butanol) to fuels is the decrease in emissions. In this regard, the role of alcohol additives consists not only of improving the energy efficiency and engineering properties of fuels but also of making them more eco-friendly and less toxic. Yet it should be also taken into account that applying various alcohols for mixing with diesel and biodiesel fuel may have several disadvantages as well. A slight decrease in several physical properties (density, viscosity, cetane number, flash point and heating value) and an increase in CO and NO_x_ emissions were reported in studies of diesel fuel blended with different alcohols [11,12,13] and natural oil esters [14,15]. Therefore, different alcohol–fuel blends should be comprehensively studied in terms of their properties to find the most versatile mixtures. For instance, it is known from the literature that biodiesel–alcohol (e.g., propanol, butanol, pentanol) blends tend to emit smaller numbers of cariogenic compounds, especially polycyclic aromatic hydrocarbons, and reduce the wet stacking in diesel engines in comparison with pure diesel. El-Seesy et al. [16] determined the optimal composition of propanol–decanol–Jatropha oil biodiesel in terms of its properties and for its use in an engine: lower viscosity and pollution level and higher cylinder pressure and heat release rate. Atmanli [11] carried out comparative analyses of diesel–waste oil biodiesel–alcohol (1-propanol, 1-butanol, 1-pentanol) ternary mixtures with diesel and biodiesel fuel themselves. Both physico-chemical properties, such as density and viscosity, and practical engine characteristics were discussed. It has been shown that addition of alcohols leads to a density and viscosity decrease, similar to the trends reported in [17,18]. The alcohols have been also found to slightly decrease the cetane number of the blends. This problem was also discussed in [13] in terms of adding a cetane improver to cancel out that effect.

Bioethanol being relatively more available, widespread and cheap may seem to be its considerable edge. At the same time, using higher alcohols is reported to improve several properties, especially miscibility [11,13,19]. As a result, n-butanol is also quite interesting to study in both binary mixtures with diesel [20,21] and biodiesel [22] fuel and ternary blends, e.g., n-butanol–diesel–cotton oil [23,24], n-butanol–diesel–palm oil [25], n-butanol–diesel–vegetable oil [26,27] and n-butanol–diesel–biodiesel fuel [28,29]. n-Pentanol has been also widely studied either as a fuel itself [30] or in mixtures: binary with diesel [17,31,32] and biodiesel [18] and ternary n-pentanol–diesel–biodiesel ones [33].

It should be also noted that, despite the high level of interest in the use of alcohols and natural oils for biodiesel production, most of the works in this field are dedicated only to the industrial and practical characteristics of the engines and fuels. At the same time, information on the physico-chemical properties for these kinds of systems is rather limited, especially in terms of thermodynamics and thermochemistry. Huang et al. [34] performed experimental determination of the evaporation and micro-combustion properties (evaporation rate, micro-explosion intensity, etc.) of propanol–soybean oil blended droplets at three temperatures. Bencheikh et al. [35] applied several methods of thermal analyses (DSC, TGA) as well as other physico-chemical methods to study several mixtures of waste cooking oil biodiesel with diesel and propanol. Addition of the alcohols was proved to reduce the crystallization temperature of the mixtures. Propanol was also found to improve several characteristics of the combined fuel. Saied et al. [36] studied castor oil adducts with three acid (phthalic, maleic, succinic) anhydrides. A number of physical properties were experimentally determined at different temperatures. Permittivity, dielectric loss and electrical conductivity were measured at 30 °C and 60 °C. Data on viscosity were obtained in the temperature range 30 °C–80 °C. The density of the mixtures was studied at 50 °C.

This paper is dedicated to the physico-chemical properties of three alcohols (n-propanol, n-butanol and n-pentanol) blended with oil. Earlier, we investigated liquid–liquid equilibria and excess enthalpies in multicomponent systems containing ethanol [37], propanol [38], butanol [39] and pentanol [40]. Because the observed alcohols are not only used as the additive to fuels or the biodiesel fuel itself, and, together with carboxylic acids and corresponding esters are the components of various natural oils, the alcohol–oil blends can be observed as model ones to investigate the physico-chemical properties which can be needed for industrial application. In this work, we present experimental results on the excess enthalpies for pseudo-binary mixtures of sunflower oil with three alcohols (n-propanol, n-butanol, n-pentanol) at 303.15 K and atmospheric pressure.

## 2. Results

**Figure 1:**
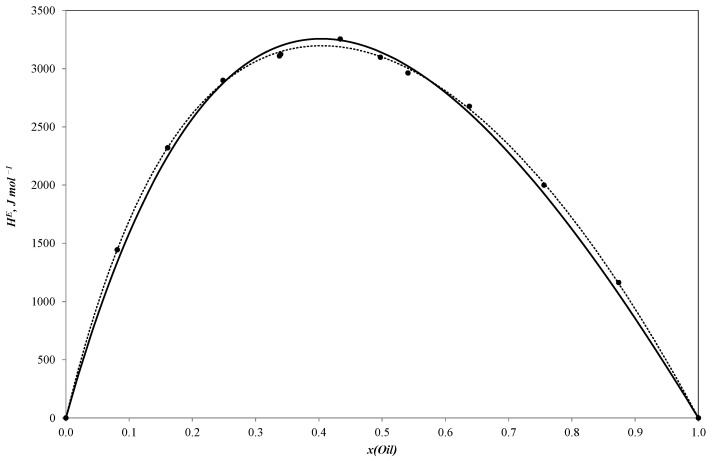
Molar excess enthalpies for binary system sunflower oil–n-propanol (J mol^−1^). The experimental solid circles (●) at 303.15 K were calculated by Redlich–Kister equation (**^……^**) and NRTL model (**―**); *x*—mole fraction of sunflower oil.

**Figure 2:**
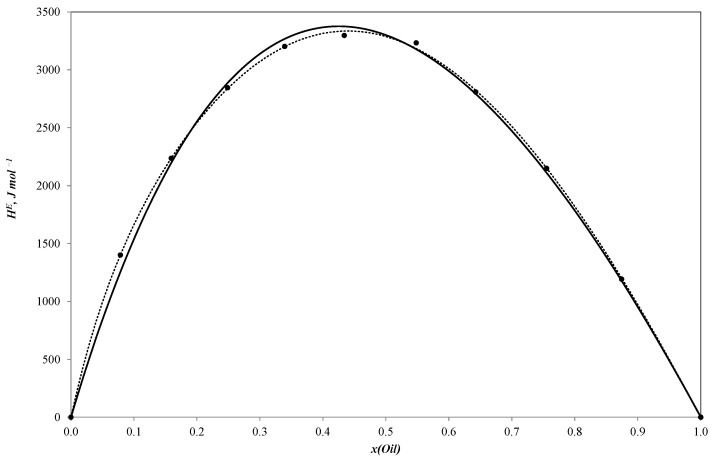
Molar excess enthalpies for binary system sunflower oil–n-butanol (J mol^−1^). The experimental solid circles (●) at 303.15 K were calculated by Redlich–Kister equation (**^……^**) and NRTL model (**―**); *x*—mole fraction of sunflower oil.

**Figure 3:**
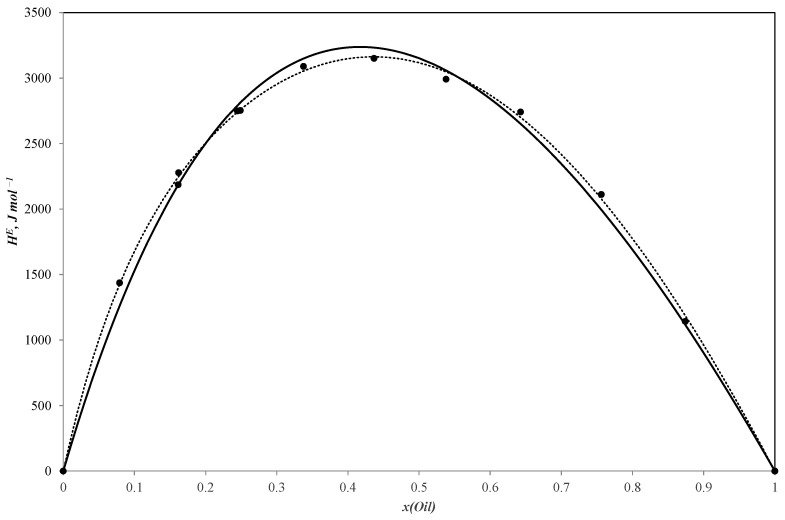
Molar excess enthalpies for binary system sunflower oil–n-pentanol (J mol^−1^). The experimental solid circles (●) at 303.15 K were calculated by Redlich–Kister equation (**^……^**) and NRTL model (**―**); *x*—mole fraction of sunflower oil.

Table 1, Table 2 and Table 3 present the new experimental results for the binary systems of sunflower oil–n-propanol, sunflower oil–n-butanol and sunflower oil–n-pentanol. These data are also visually represented in Figure 1, Figure 2 and Figure 3.

## 3. Discussion

All three curves of the molar excess enthalpy for the oil–alcohol (n-propanol, n-butanol, n-pentanol) pseudo-binary mixtures have a similar shape with a maximum of 3255.2 J/mole for the n-propanol–oil blend, 3297.4 J/mole for the n-butanol–oil mixture and 3150.1 J/mole for the n-pentanol–oil system. As these alcohols are completely miscible with sunflower oil, the $H_{m}^{E}$ curves are continuous ones with molar excess enthalpy values remaining positive throughout the entire concentration range. The positive values tend to be reliable as the mixing process is expected to be exothermal.

It should be noted that the molar excess enthalpy values for the three systems are quite similar. The curves are consequently near to each other, which means that the heat effect of mixing tends to weakly depend on the number of carbon atoms in the alcohol molecule. This effect may be explained in terms of the NRTL (non-random two-liquid) approach [41], which assumes that the molar excess Gibbs energy for a binary solution is the sum of two changes in residual Gibbs energy: of transferring molecules from the local cells in their pure liquids to the corresponding cells in the solution. We may expect the same relation to hold true for enthalpy on a qualitative level. Thus, we can consider the molar excess enthalpy as the sum of the contribution that corresponds to transferring every component from the pure liquid to the solution. As a result, if the contribution of the alcohol is much lower than those of the oil components, or if they are comparable for every oil–alcohol mixture, the difference in the corresponding contributions for different alcohols should not considerably affect the total molar excess enthalpy of the systems.

We have conducted comparative data analyses. Unfortunately, there are no similar studies in the literature, and the number of related studies is extremely limited. However, we have identified several relevant works. For instance, in the study by Abbas and Gmehling [42], data on excess enthalpies for binary alcohol–ketone systems were obtained. The authors also provided data from the literature on excess enthalpies of alcohols such as 2-butanol and tret-butanol. Additionally, Domańska et al. [43] investigated phase equilibria and excess molar enthalpies in binary systems involving pyrrole + hydrocarbon or an alcohol. They presented experimental data on excess enthalpies in systems such as pyrrole + 1-propanol, 1-butanol or 1-pentanol. Although the systems studied differ from ours, the findings shed light on the thermochemical behavior of alcohol mixtures.

**Figure 4:**
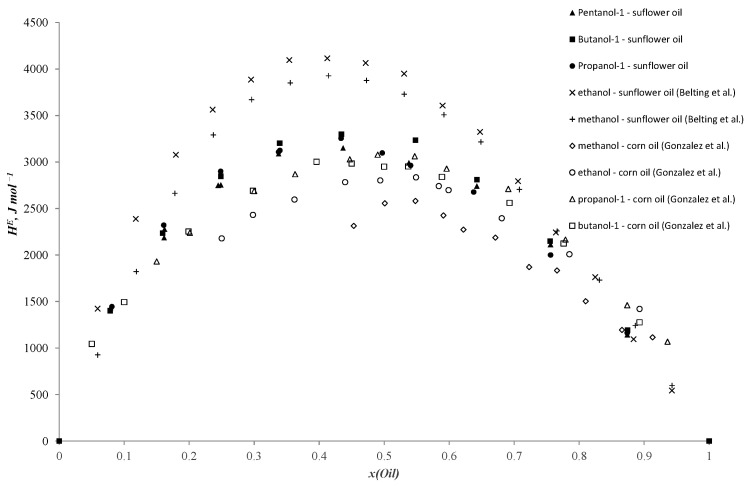
Molar excess enthalpies for binary systems: sunflower oil–n-propanol (●), sunflower oil–n-butanol (■), sunflower oil–n-pentanol (▲) experimental data at 303.15 K vs. sunflower oil–methanol (+) (353.15 K) [46], sunflower oil–ethanol (×) (353.15 K) [46], corn oil–methanol (◇) (298.15 K) [44], corn oil–ethanol (◯) (298.15 K) [44], corn oil–n-propanol (△) (298.15 K) [44], corn oil–n-butanol (□) (298.15 K) [44] (J mol^−1^).

Works [44,45,46] are more relevant to our study. González et al. [44] investigated enthalpies of mixing in binary systems of alcohols and n-alkanes with corn oil. In particular, they present experimental data on excess enthalpies in methanol, ethanol, propanol-1, propanol-2, butanol-1 and butanol-2 + corn oil systems. In Figure 1 of the study [44], data are presented illustrating a trend of increasing excess enthalpies with the elongation of the carbon chain in alcohols. However, it is noteworthy that, in the systems of 1-propanol and 1-butanol + corn oil, the thermal effects are very similar, and the dependence curves lie closely to each other. Similar results are observed in the current investigation. These primary alcohols (1-propanol, 1-butanol) exhibit similar polarity and consequently yield comparable values of mixing enthalpies. For shorter alcohols like methanol and ethanol, the polarity values are higher due to the significant induction effect in ethanol compared to methanol, which varies as the length of the hydrocarbon chain increases. However, the contribution of each subsequent carbon atom decreases. Similar conclusions are drawn by the authors in another publication [45]. Belting et al. [46] provide data on mixing enthalpies in binary systems of methanol and ethanol + sunflower oil. It is noteworthy to highlight the interesting findings of this study regarding the mixing enthalpies in the ethanol + sunflower oil system as this system exhibits limited solubility between its components, and the results are obtained across the entire concentration range. The authors mention that they did not observe stratification in this system at higher temperatures. Additionally, the authors of the article drew attention to how variations in experimental temperature influence thermal effects, which we can also observe from the experimental results (the mixing enthalpy values measured at other temperatures are higher than the rest of the dataset). Comparison graphs for the data from the literature and experimental results of the current study are presented in Figure 4.

Both the NRTL equation and Redlich–Kister equation were shown to be in sufficient agreement with the obtained values, which is why they could be used for the data correlation and interpolation. In general, the Redlich–Kister equation with the exponential switching function was found to give a better result than the NRTL equation. At the same time, the chosen Redlich–Kister equation uses more adjustable parameters for data correlation.

## 4. Materials and Methods

### 4.1. Materials

For the investigation, n-propanol, n-butanol and n-pentanol provided by Vekton (Saint Petersburg, Russia) were taken. Preliminary purification of alcohols was carried out by drying over molecular sieves (zeolites with a pore diameter of 3 Å). The purity of dried reagents was checked by gas chromatography (GC) method using “CHROMATEC CRYSTAL 5000.2” (Yoshkar Ola, Russia) chromatograph with a thermal conductivity detector (TCD) equipped with a Hayesep Q 80/100 packed column (3 m × 2 mm). The standard uncertainty of GC analysis is ±0.005 mole fraction. The final purities of chemicals are presented in Table 4. The sunflower oil used in this study was from a local commercial supplier (Saint Petersburg, Russia, GOST 1129-2013).

### 4.2. Solubility Measurements

Before conducting the molar excess enthalpy measurements, the investigation of the solubility of binary alcohol–oil systems was carried out. The measurements were performed by the titration method using “cloud point” technique [47]. The analysis showed that n-propanol–oil, n-butanol–oil and n-pentanol–oil systems remain homogeneous throughout the entire concentration range.

### 4.3. The Kinetics of the Transesterification Reaction Investigation

The kinetics of this reaction were studied to ensure that no chemical reaction (transesterification) occurred between the oil components and alcohols during the experiment. To confirm the accuracy of the measured excess enthalpies in the sunflower oil–alcohol (n-propanol, n-butanol, n-pentanol) systems, it was necessary to exclude the influence of interfering factors on the measurement results, primarily chemical reactions.

Based on general chemical considerations, it can be assumed that transesterification reactions of triglyceride with alcohols, leading to the formation of diglyceride and esters of fatty acid in accordance with Figure S7, may potentially occur. Moreover, nucleophilic addition of an alcohol to the double bond of unsaturated fatty acids and other reactions involving minor components of sunflower oil can possibly take place in the investigated systems.

Although the mentioned reactions usually take place under catalytic conditions, we decided to conduct the experiment to demonstrate that these reactions do not occur significantly in the investigated mixtures, and, in this case, their presence did not affect the course of the experiment.

To achieve this, solutions of 200 mg of sunflower oil in 2 mL of each investigated alcohol (n-propanol, n-butanol, n-pentanol) were prepared. The alcohol was taken in a significant excess to maximize the acceleration of possible chemical reactions. NMR spectra were recorded on ^1^H nuclei (with accumulation) for each of these mixtures. After that, the mixtures were kept at a temperature of 60 °C for 4 h. The experimental temperature significantly exceeded the temperature of excess enthalpy measurements to maximize the acceleration of possible chemical reactions. After cooling the mixtures to ambient temperature, NMR spectra were recorded again on ^1^H nuclei (with accumulation).

A detailed comparison of the spectra of the mixtures before and after heating was carried out for each alcohol. Particular attention was given to the region of 3.7–4.5 ppm, where the appearance of a signal from the CH_2_ group corresponded to formation of the ester of alcohol and fatty acid (the second product in Figure 4) or addition products.

Figure S1 shows the comparison of the spectra of the sunflower oil solution in n-propanol before heating (spectrum B) and after heating (spectrum A). The positions of the n-propanol signals are indicated (they exceed the scale in this case), as well as their ^13^C satellites, the intensity of which is significant due to the large excess of alcohol. The integrals of the characteristic signals of the components of sunflower oil are indicated. It was revealed that the spectra were identical within the experimental error, and there were no new signals in the region of 3.7–4.5 ppm. Similar results were observed for the solutions of sunflower oil in n-butanol and n-pentanol.

Figure S2 shows the comparison of the spectra of heated solutions of sunflower oil in n-butanol (spectrum A) and n-pentanol (B) with pure oil (spectrum C). Spectra A and B showed intense signals from various groups in the alcohol molecule and their ^13^C satellites. Spectrum C indicates the positions of characteristic groups: CH_2_-glyc, CH-glyc–protons of the glycerol residue, CH-unsat–protons near double bonds and CH_2_-linoleic–protons of the CH_2_ group in the linoleic acid residue, which are located between double bonds and the CH_3_ end terminal group of fatty acid residues. It was demonstrated that there were no new signals of noticeable intensity in the spectra of the solutions except the signals of triglyceride and alcohol.

The ^1^H NMR spectra of the mixtures before and after heating were found to be identical. No new signals, distortions of the shape or changes of integral values of existing signals were observed. Considering that the temperature and heating time significantly exceeded those during the measurement of the excess enthalpies, it can be concluded that negligible chemical transformations took place, and the resulting changes in values did not exceed the instrument’s error range. Thus, the excess enthalpies of sunflower oil with alcohols, namely n-propanol, n-butanol and n-pentanol, were accurately measured without the systematic error introduced by chemical reaction.

### 4.4. Molar Mass of Sunflower Oil

To enhance the visual representation of results and enable a more precise analysis of the substances investigated in this study, we scrutinized the sunflower oil to determine its molecular mass. The mean molecular mass of the oil under study was determined by means of electrospray ionization mass spectroscopy (ESI-MS). The advantage of this method is the use of the so-called “soft ionization” technique. The forming of pseudo molecular ions in ESI-MS allows the structure of the initial molecule to be saved without significant oxidation, fragmentation, etc. ESI-MS is a common practice method for the study of a rather wide range of compounds with molecular mass 1–7 kDa [48]. The obtained mass spectrum of the oil studied in this work is presented in the Figure S3. From the most valuable signals of the spectra, with respect to the contribution (ratio) each of them, the mean molar mass of the oil under consideration was estimated to be equal to 1138 g/mol.

### 4.5. Molar Excess Enthalpy Measurements

In the experiment, an isothermal calorimeter Setaram C80 (Caluire, France) was utilized. The temperature measurement accuracy was ±0.1 K, sensitivity was 30 μV/mW, signal resolution was 0.1 μW and noise level was 1 μW. Calibration was performed using the Joule effect, and temperature calibration was carried out using standard samples.

Membrane mixing cells, consisting of two parts separated by a thin aluminum membrane, were employed for measuring the heat of mixing. The weighing method on Sartorius MSU225S (Goettingen, Germany) balances with an accuracy of ±0.1 mg was used to determine the substance quantities. In the process of the experiment, substances were mixed using a reversible mechanism, ensuring complete mixing without additional thermal impact. Each experimental mixture was studied in two cells alternately. Additional details of the methodology can be found in references [37,49]. After the experiment, the used samples were kept for 24 h at 303.15 K. During this time, no phase separation of the solutions occurred, indicating that there were no reaction or phase processes in them.

Calorimetric signal stabilization was conducted before the start of the experiment, after which the components were mixed, and the thermal effect was recorded. The process concluded when the signal stabilized for 30 min. To account for the membrane rupture effect, a blank drop of the rod was performed. Examples of obtained thermograms are presented in Figures S4–S6. The heat effects were calculated by integrating the peaks of the heat flow signal over time in the Calisto program.

### 4.6. Calculation

#### 4.6.1. Redlich–Kister

Obtained experimental data were correlated with use of the improved Redlich–Kister equation [50,51] in order to check their values for thermodynamic correspondence.

(1) $$H^{E}=x_{A}x_{B}\left(S\sum_{i=0}^{N}a_{i}{\left(x_{A}-x_{B}\right)}^{i}+\left(1-S\right)\sum_{i=0}^{M}b_{i}{\left(x_{A}-x_{B}\right)}^{i}\right)$$

where $x_{A}{,x}_{B}$ are the molar fractions of components *A* and *B*, $a_{i}$ and $b_{i}$ are the adjustable parameters, *N* and *M* are the polynomial degree and S is the switching function. The improved formula of the Redlich–Kister equation with exponential switching function was found to correlate all experimental data in the best way.

(2) $$S=exp\left(-\gamma x_{a}\right)$$

The simulation results are shown in Figure 1, Figure 2 and Figure 3.

To characterize the best description of the Redlich–Kister equation by a polynomial for a set of experimental points, the standard deviation parameter was used.

(3) $$\sigma (H^{E})=\sqrt{\frac{\sum_{i=1}^{n}{\left(H_{calc,i}^{E}-H_{exp,i}^{E}\right)}^{2}}{n-N}}$$

where *n* is the number of experimental points, *N* is the number of coefficients of the polynomial. The average calculation error was estimated using the following formula:

(4) $$ARD\left(\%\right)=\frac{100}{n}\sum_{i=1}^{n}\frac{\left|H_{calc,i}^{E}-H_{exp,i}^{E}\right|}{\left|H_{exp,i}^{E}\right|}$$

Parameters of these equations, average relative deviation (ARD) and standard deviation ($\sigma \left(H^{E}\right)$) are presented in Table 5.

#### 4.6.2. Non-Random Two-Liquid Model

The NRTL model [41] was used to approximate experimental results on the enthalpies of mixing binary systems as follows:

(5) $$H^{E}=x_{1}x_{2}\left[\frac{G_{21}∆g_{21}\left(x_{1}+x_{2}G_{21}-x_{1}\tau_{21}\alpha_{12}\right)}{{\left(x_{1}+x_{2}G_{21}\right)}^{2}}+\frac{G_{12}∆g_{12}\left(x_{2}+x_{1}G_{12}-x_{2}\tau_{12}\alpha_{12}\right)}{{\left(x_{1}+x_{2}G_{21}\right)}^{2}}\right],$$

where

(6) $$G_{12}=exp\left(-\alpha_{12}\tau_{12}\right),\;G_{21}=exp\left(-\alpha_{12}\tau_{21}\right),\;\tau_{12}=\frac{∆g_{12}}{RT},\;\tau_{21}=\frac{∆g_{21}}{RT},\;G_{12}=exp\frac{∆g_{12}}{RT},$$

where $∆g_{12}=g_{12}-g_{22}$ and $∆g_{21}=g_{21}-g_{11}$ are adjustable binary parameters, and *α*_12_ is the non-randomness parameter.

When finding the coefficients of the equation, the objective function, OF, was minimized as follows:

(7) $$OF=\sum_{i=1}^{n}{\left(\frac{H_{calc,i}^{E}-H_{exp,i}^{E}}{H_{exp,i}^{E}}\right)}^{2}$$

where the summation is over all *i* data points.

Parameters of the NRTL model and ARD values (calculated with Equation (4)) are given in Table 6 and plotted in Figure 1, Figure 2 and Figure 3.

Both the Redlich–Kister and the NRTL equations have been found to correlate experimental data on the molar excess enthalpy with sufficient accuracy. The average relative deviation of the calculations (0.8%–1.4% for the Redlich–Kister equation and 3% for the NRTL equation) tends to be similar to the experimental uncertainty. It can be seen that the used form of the Redlich–Kister equation fits the experimental values more precisely due to the fact that it contains more adjustable parameters.

## 5. Conclusions

This study investigated the thermochemical properties of biofuel components, focusing on the potential of alcohols (n-propanol, n-butanol, n-pentanol) as “green” alternatives blended with sunflower oil. Calorimetric analysis at 303.15 K allowed for the experimental determination of excess enthalpies in pseudo-binary mixtures. Despite structural differences in alcohols, molar excess enthalpy values display uniformity. The similarity in molar excess enthalpy values across the different alcohol blends implies weak dependence on the number of carbon atoms in the alcohol molecule. Thus, the peak values of excess enthalpies for systems of sunflower oil with n-propanol, n-butanol and n-pentanol were, respectively, 3255.2 J/mole, 3297.4 J/mole and 3150.1 J/mole. Both the NRTL and Redlich–Kister equations exhibited agreement with the obtained values. The Redlich–Kister equation with an exponential switching function provides better results, although it employs more adjustable parameters for data correlation. To ensure the accuracy of excess enthalpy measurements, the study confirmed the chemical stability of the sunflower oil–alcohol systems. NMR spectra comparisons before and after heating demonstrated the absence of significant chemical transformations during the experimental period. Due to a lack of reported data on measuring excess enthalpies for such systems in the literature, direct comparisons with existing results are limited. The study contributes valuable insights into the physico-chemical properties of alcohol–oil systems, particularly in terms of thermodynamics and thermochemistry. This work lays the foundation for further research on the physico-chemical properties of alcohol–oil systems. For a comprehensive picture of the behavior of oil–alcohol systems, it is necessary to carry out more investigations, covering the entire temperature range of 293.15 K–323.15 K. Such conditions correspond to the principles of energy-saving chemical technologies. In addition, studies are also required on, in particular, the excess enthalpies for mixtures of ethanol–sunflower oil and hexanol–sunflower oil under various conditions. The results of these studies will make it possible to evaluate the patterns of behavior of the alcohol mixtures that are so important for use in bioenergy as additives to biodiesel since they increase its energy efficiency and improve its technical properties while making the fuel more environmentally friendly.

## Figures and Tables

**Table 1:** Molar excess enthalpies of the oil + n-propanol system at 303.15 K ^a^ (J mol^−1^), *x*—mole fraction of oil.

*x* (Oil)	$H_{m}^{E}$/J mol^−1^	*x* (Oil)	$H_{m}^{E}$/J mol^−1^
0.0811	1444.5	0.4971	3098.1
0.1609	2320.9	0.5408	2962.9
0.2484	2899.7	0.6377	2677.5
0.3375	3109.0	0.7559	2000.1
0.3396	3124.3	0.8740	1162.9
0.4337	3255.2		

^a^ Standard uncertainties of temperature *u*(*T*) = 0.05 K, mole fraction *u*(*x*) = 0.0001 and molar excess enthalpies is *U_r_*($H_{m}^{E}$) = 0.03 (95% level of confidence).

**Table 2:** Excess enthalpies of the oil + n-butanol system at 303.15 K ^a^ (J mol^−1^), *x*—mole fraction of oil.

*x* (Oil)	$H_{m}^{E}$/J mol^−1^	*x* (Oil)	$H_{m}^{E}$/J mol^−1^
0.0785	1401.7	0.6427	2809.3
0.1592	2236.5	0.7554	2149.3
0.2489	2846.0	0.8745	1193.1
0.3394	3202.5		
0.4340	3297.4		
0.5483	3232.7		

^a^ Standard uncertainties of temperature *u*(*T*) = 0.05 K, mole fraction *u*(*x*) = 0.0001 and molar excess enthalpies is *U_r_*($H_{m}^{E}$) = 0.03 (95% level of confidence).

**Table 3:** Excess enthalpies of the oil + n-pentanol system at 303.15 K ^a^ (J mol^−1^), *x*—mole fraction of oil.

*x* (Oil)	$H_{m}^{E}$/J mol^−1^	*x* (Oil)	$H_{m}^{E}$/J mol^−1^
0.0790	1437.1	0.4367	3150.1
0.1614	2186.8	0.5379	2991.0
0.1620	2278.4	0.6426	2741.5
0.2446	2749.7	0.7560	2112.4
0.2488	2753.0	0.8742	1142.8
0.3377	3089.6		

^a^ Standard uncertainties of temperature *u*(*T*) = 0.05 K, mole fraction *u*(*x*) = 0.0001 and molar excess enthalpies is *U_r_*($H_{m}^{E}$) = 0.03 (95% level of confidence).

**Table 4:** The purities of the used chemicals.

	Substance	Symbolic Name	Source	Purity, Mole Fraction	Purification Method	Analysis Technique
71-23-8	n-Propanol	PrOH	Vekton (Russia)	0.998 ^b^	Drying	GC ^a^
71-36-3	n-Butanol	BuOH	Vekton (Russia)	0.995 ^b^	Drying	GC ^a^
71-41-0	n-Pentanol	AmOH	Vekton (Russia)	0.997 ^b^	Drying	GC ^a^
8001-21-6	Sunflower seed oil	-	Local commercial supplier	-	-	-

^a^ Gas chromatography. ^b^ Standard uncertainties of mole fraction *u*(*x*) = 0.005.

**Table 5:** Fitting parameters *a_k_* for Equations (1) and (2) for binary mixtures of sunflower oil–alcohols with ARD and standard deviations, *σ* ($H_{m}^{E}$, J mol^−1^).

	Oil + n-Propanol	Oil + n-Butanol	Oil + n-Pentanol
*a*_0_	22,278.57	24,733	25,455.93
*a*_1_	−89.89	−97.49	−90.94
*b*_0_	10,854.76	13,109.95	12,252.04
*b*_1_	−995.00	−2888.47	−1972.92
*γ*	4.01	11.22	8.09
ARD (%)	0.8	0.5	1.4
*σ*, J mol^−1^	34	26	42

**Table 6:** Binary interaction parameters of the NRTL model for binary mixtures of sunflower oil–alcohols.

	Oil(1) + n-Propanol(2)	Oil(1) + n-Butanol(2)	Oil(1) + n-Pentanol(2)
∆*g*_12_	6002.02	7250.13	48,841.79
∆*g*_21_	−7979.14	−7862.53	28,104.44
*α_ij_*	−0.14	−0.11	0.06
ARD (%)	3	3	3

## Data Availability

Data are contained within the article and Supplementary Materials.

## Supplementary Materials

The following supporting information can be downloaded at: https://www.mdpi.com/article/10.3390/ijms25063244/s1.
