# Supplementary material for: Excess Enthalpies Analysis of Biofuel Components: Sunflower Oil–Alcohols Systems
Source: Int J Mol Sci. 2024 Mar 13;25(6):3244. doi: 10.3390/ijms25063244 (PMC10970517; doi:10.3390/ijms25063244)
Supplement: Supplementary file 1 [file ijms-25-03244-s001.zip › ijms-2866800-supplementary.pdf]

Supplementary materials

Excess Enthalpies Analysis of Biofuel Components: Sunflower Oil - Alcohols Systems

Alexandra Golikova *, Anna Shasherina, Yuri Anufrikov, Georgii Misikov, Petr Kuzmenko, Alexander Smirnov, Maria Toikka and Alexander Toikka

Institute of Chemistry, St. Petersburg State University, Universitetskiy Prospect 26, Peterhof, Saint Petersburg 198504, Russia

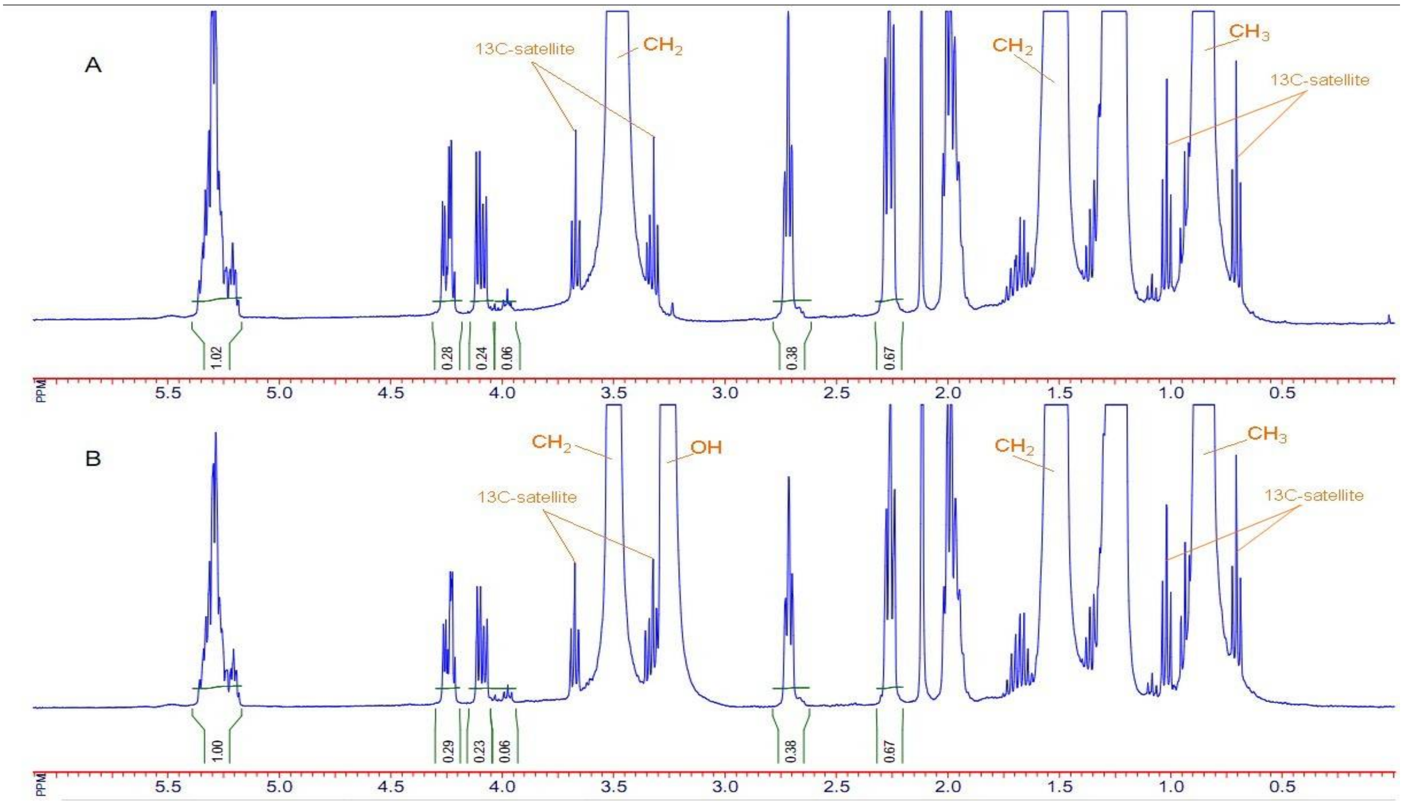

Figure S1. ^{1}H NMR spectra comparison of the sunflower oil – n-propanol solution before (B) and after (A) heating

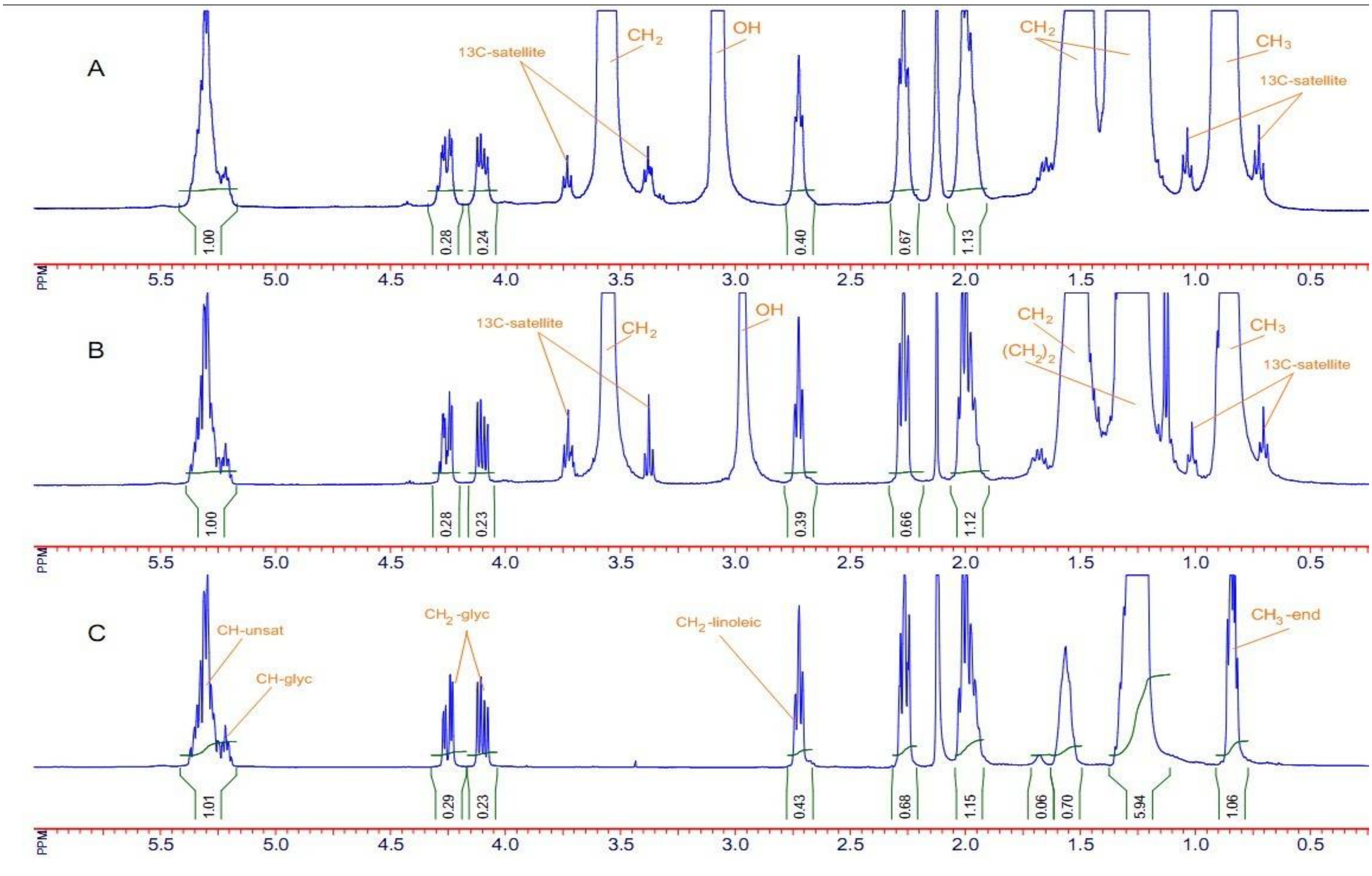

Figure S2. ^{1}H NMR spectra comparison of the pure sunflower oil (C) and sunflower oil – n-butanol (A), sunflower oil – n-pentanol (B) systems after heating

Display Report

Analysis Info

Analysis Name	D:\Data\Work\2023\11\15\00-.d	Acquisition Date	15-Nov-23 13:04:50
Method	20230815_tune_high_neg.m	Operator	BDAL@DE
Sample Name	BuOH	Instrument / Ser#	maXis 62
Comment			

Acquisition Parameter

Source Type	ESI	Ion Polarity	Negative	Set Nebulizer	0.8 Bar
Focus	Not active			Set Dry Heater	180 °C
Scan Begin	50 m/z	Set Capillary	5500 V	Set Dry Gas	4.0 l/min
Scan End	3000 m/z	Set End Plate Offset	-500 V	Set Divert Valve	Source

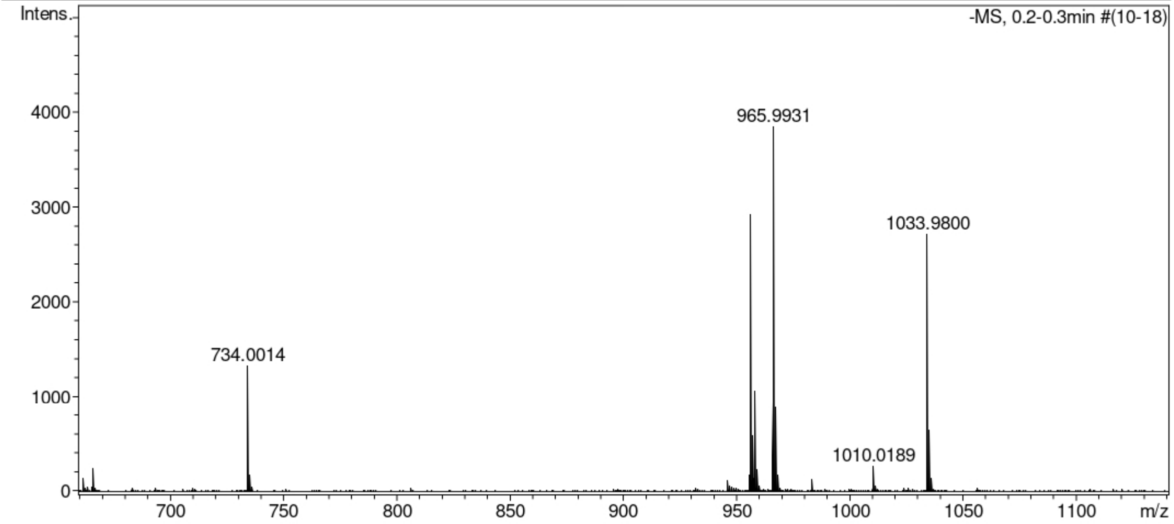

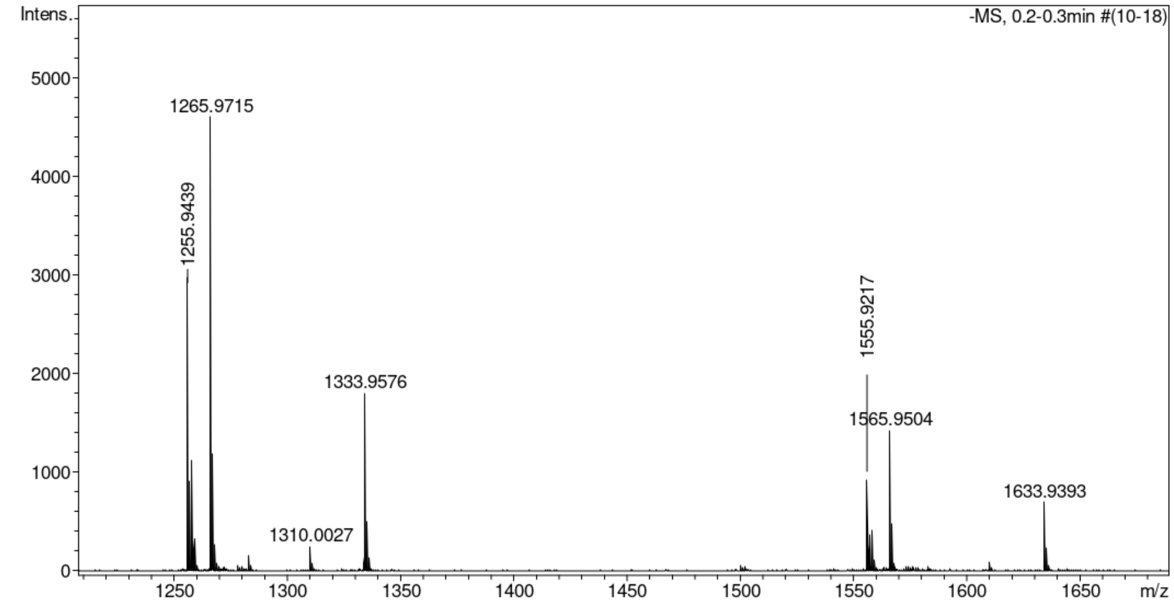

Figure S3. Mass-spectrum of the oil under study obtained by ESI-MS

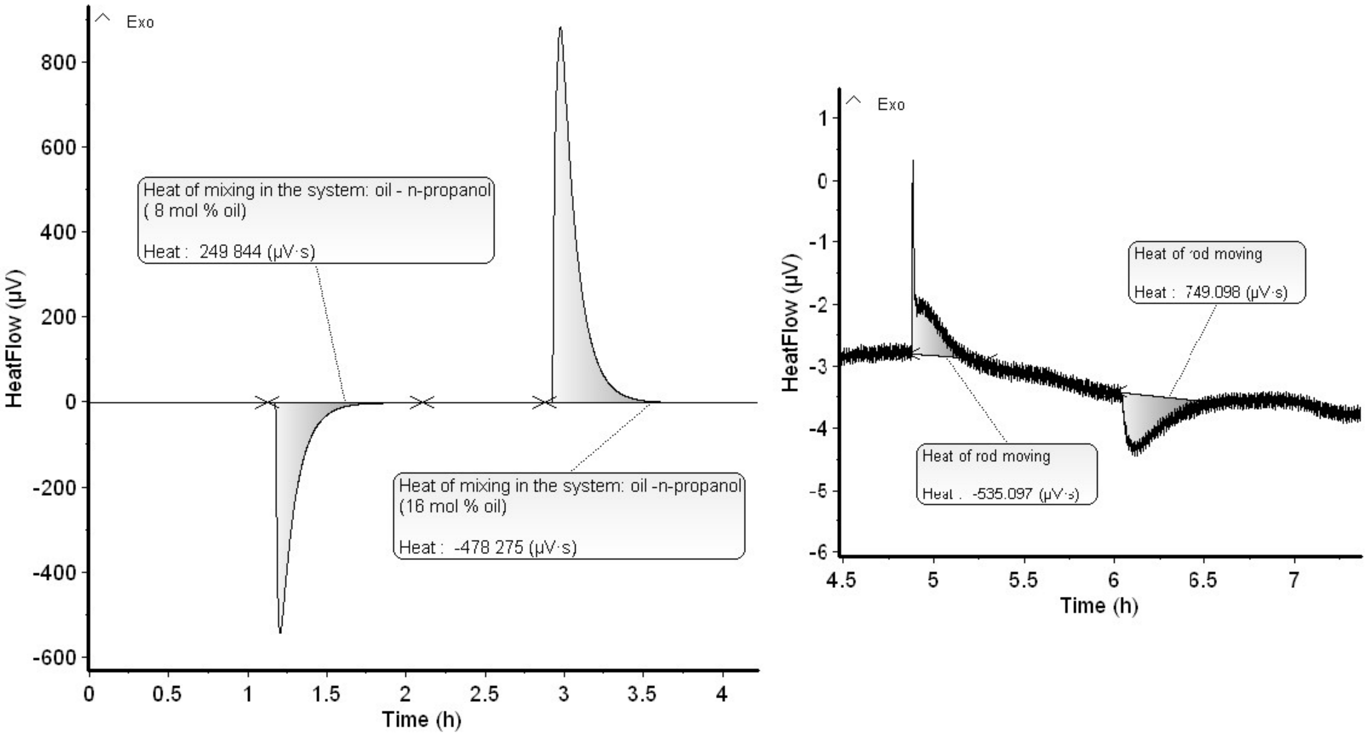

Figure S4. Example of a signal obtained on a C80 calorimeter and its processing in the Calisto program (sunflower oil – n-propanol system)

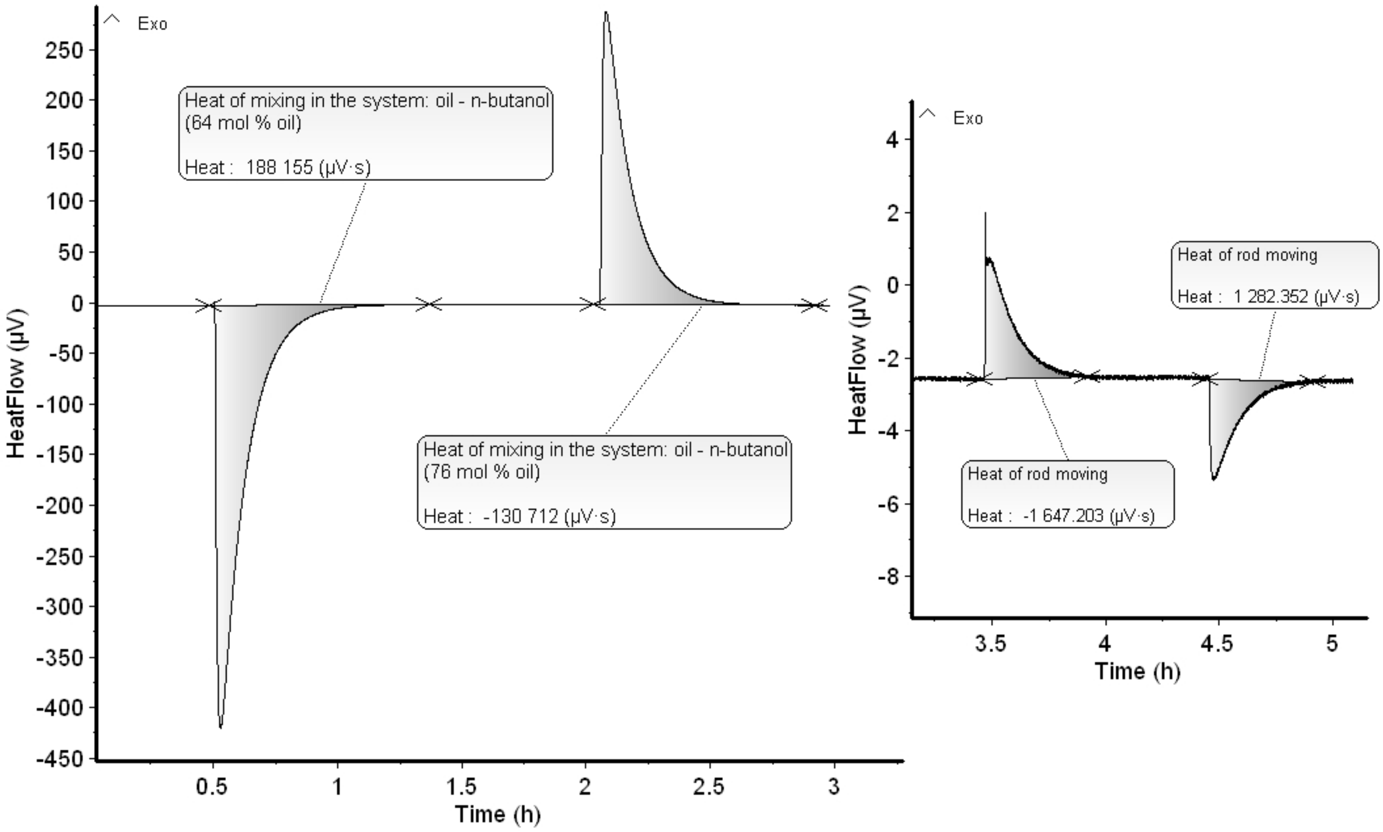

Figure S5. Example of a signal obtained on a C80 calorimeter and its processing in the Calisto program (sunflower oil – n-butanol system)

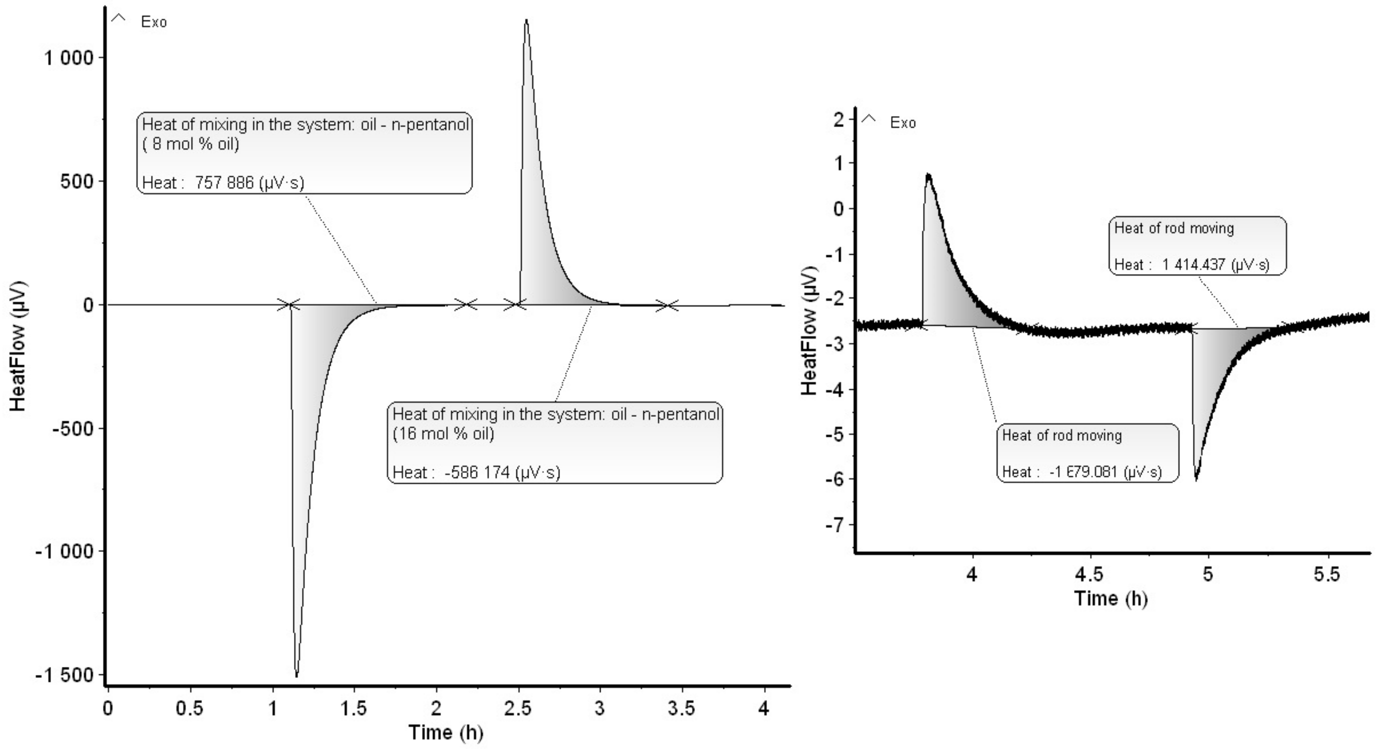

Figure S6. Example of a signal obtained on a C80 calorimeter and its processing in the Calisto program (sunflower oil – n-pentanol system)

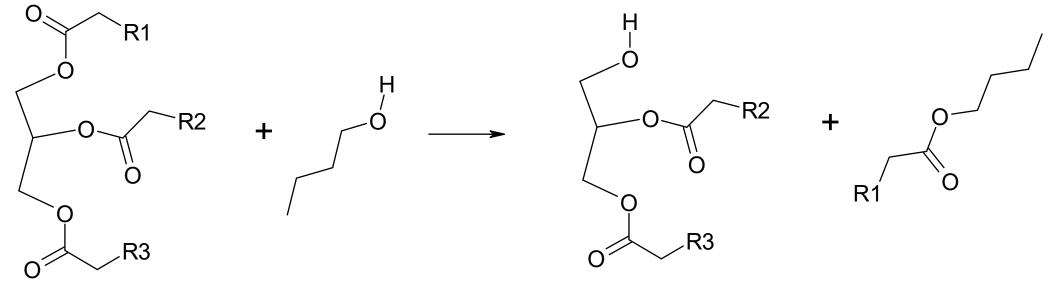

Figure S7. Transesterification reaction of triglyceride with alcohols
